# Application of conventional and contrast-enhanced ultrasound in diagnosing cervical tuberculous lymphadenitis

**DOI:** 10.3389/fmed.2026.1797878

**Published:** 2026-06-17

**Authors:** Jun Hu, Wenzhi Zhang, Peijun Chen, Ying Zhang, Gaoyi Yang

**Affiliations:** 1 Department of Ultrasonography, Hangzhou Red Cross Hospital, Zhejiang Hospital of Integrated Traditional Chinese and Western Medicine, Hangzhou, Zhejiang, China; 2 Department of Ultrasonography, Affiliated Hangzhou First People’s Hospital, School of Medicine, Westlake University, Hangzhou, Zhejiang, China

**Keywords:** contrast-enhanced ultrasound, logistic regression model, lymph node, tuberculous lymphadenitis, ultrasound

## Abstract

**Objective:**

To evaluate the predictive value of contrast-enhanced ultrasonography (CEUS) in differentiating cervical tuberculous lymphadenitis (CTL) from non-tuberculous cervical lymphadenitis (NCTL).

**Methods:**

A retrospective analysis was conducted on 303 patients with cervical lymphadenopathy. Various CEUS features, including the long axis diameter (L), short axis diameter (S), length-to-width ratio (L/S), presence of the hilum, boundary clarity, internal echogenicity, calcification, necrosis, blood flow distribution within the lymph node, and direction of blood flow, were examined. A multivariate logistic regression model was developed to predict CTL.

**Results:**

The model identified key predictive factors, including absent hilum, necrosis, heterogeneous enhancement, perfusion defects, peripheral ring enhancement, and non-centrifugal enhancement. In the training cohort (*n* = 202), the model achieved an AUC of 0.913, with high specificity (77.5%) and sensitivity (92.6%). Similar performance was observed in the validation cohort (*n* = 101, AUC = 0.917).

**Conclusion:**

CEUS analysis demonstrates significant efficacy in distinguishing CTL from NCTL, thereby enhancing diagnostic accuracy.

## Introduction

Tuberculosis (TB), caused by *Mycobacterium tuberculosis* (MTB), is a chronic infectious disease. The COVID-19 pandemic has had a significant negative impact on the diagnosis and care of tuberculosis, causing it to once again become the leading cause of death from a single pathogen globally (1). As a nation with a high TB burden, China continues to face considerable challenges in the prevention and control of this disease (2, 3). Among the various forms of extra-pulmonary TB, superficial lymph node tuberculosis (LNTB) is the most common prevalent, accounting for up to 50% of cases, with the cervical region showing a particularly high prevalence (4–6). The early clinical manifestations of cervical lymphadenitis are often nonspecific, and laboratory and imaging tests demonstrate low sensitivity, leading to frequent misdiagnoses and delays in diagnosis. Such delays can result in significant lymph node enlargement, abscess formation, and the development of sinus tracts, which can severely affect the patient’s appearance and social life, thus significantly impairing their quality of life (7).

In recent years, contrast-enhanced ultrasonography (CEUS) has become increasingly applied in the diagnosis and management of cervical tuberculous lymphadenitis (CTL). In comparison to conventional ultrasonography (US), including two-dimensional ultrasound and color Doppler ultrasonography, CEUS offers superior sensitivity for detecting microvascular structures, enabling real-time visualization of microvascular perfusion. This technique facilitates an effective evaluation of blood perfusion within tissues and partial microcirculation in solid organs (8, 9).

The objective of this study is to explore the predictive value of a CEUS-based qualitative and quantitative analysis model in distinguishing CTL from non-tuberculous cervical lymphadenitis (NCTL), with the aim of improving diagnostic accuracy in differentiating between CTL and NCTL.

## Materials and methods

### Patients

A retrospective analysis was conducted on all patients with cervical lymphadenopathy treated at Hangzhou Red Cross Hospital between January 2022 and June 2023. This study was approved by the Ethics Committee of Hangzhou Red Cross Hospital (No. 2022-112), and informed consent was obtained from all participants. Cases were screened according to inclusion and exclusion criteria. The inclusion criteria were as follows: (1) presence of enlarged lymph nodes in the neck, (2) confirmation of lymph node tuberculosis through pathological or laboratory examination, (3) absence of allergic constitution and ability to tolerate CEUS examination, (4) absence of any otherunderlying diseases and ability to undergo needle biopsy, and (5) availability of comprehensive imaging data, including conventional ultrasound, CEUS, and needle biopsy, obtained during the initial diagnosis at Hangzhou Red Cross Hospital. Furthermore, participants needed to give consent for their data to be used in the analysis of this study. The exclusion criteria were: (1) unclear clinical diagnosis; (2) prior treatment with medication; (3) poor quality of US or CEUS images, or if the CEUS video was too brief or failed to maintain continuous focus on the target lymph node.

The Composite Reference Standard (CRS) was adopted, based on the comprehensive TB guidelines from the World Health Organization and the clinical diagnostic guidelines for TB from the National Institute for Health and Care Excellence (NICE). The diagnostic criteria were as follows: (1) clinical symptoms and imaging (ultrasound, CT, MRI) consistent with CTL; (2) pathological examination of tissue samples indicative of CTL; (3) laboratory tests, such as MTB culture, confirming infection with MTB; (4) effective treatment or regular anti-tuberculosis therapy for over 6 months, with corresponding clinical symptom relief or significant improvement. If two or more of these criteria were met, the diagnosis of LNTB was confirmed.

### Ultrasound examination

The ultrasound diagnostic equipment used in this study was the Resona 7S color Doppler ultrasound system manufactured by Mindray (Shenzhen, China), equipped with L12-5 (5–12 MHz) and L9-3U (3–9 MHz) high-frequency linear array probes. Patients were positioned either supine or in the lateral decubitus position to fully expose the cervical region. US employed the L12-5 probe to assess lymph node characteristics, including location, shape, size, internal echogenicity, vascularity, and anatomical relationships with adjacent tissues. Clear, high-resolution images were selected to observe and record the maximum transverse section of the lymph node. Parameters evaluated included: the long axis diameter (L), short axis diameter (S), the length-to-width ratio (L/S), presence of the hilum, the clarity of the boundary, internal echogenicity, calcification, necrosis, blood flow distribution within the lymph node, and direction of blood flow.

To minimize variability between specimen analyses, CEUS was performed prior to biopsy, with specimens taken from the enhanced regions within the lymph nodes. The L9-3U probe was used for CEUS, employing dual-frame imaging for dynamic observation of the contrast interface. The contrast agent, SonoVue (Bracco, Italy), was prepared by reconstituting the freeze-dried powder with 5.0 mL of 0.9% sodium chloride solution, followed by high-speed agitation for 20 s to form a uniform milky suspension of microbubbles. The operator administered 2.4 mL of the contrast agent via a peripheral intravenous line, initiating CEUS immediately upon injection, followed by a 5 ml flush of physiological saline. Upon the contrast agent injection, the operator promptly activated the built-in timer of the ultrasound device and meticulously monitored the imaging for a duration of 3 min, with all images stored on the device s a duration

The dynamic video footage stored on the ultrasound system was meticulously examined frame by frame to assess the homogeneity of lymph node perfusion during CEUS, as well as to identify the presence of perfusion defects and peripheral ring enhancement (10). The following criteria were applied: (1) Perfusion Homogeneity: This was categorized as either homogeneous or heterogeneous enhancement. Homogeneous enhancement indicated uniform contrast agent distribution throughout the lymph node, while heterogeneous enhancement was characterized by uneven contrast distribution. (2) Perfusion Defect: Throughout the CEUS process, if the entire lymph node demonstrated contrast agent filling, no perfusion defect was identified. Conversely, the presence of non-perfused areas within the lymph node indicated a perfusion defect. (3) Peripheral Ring Enhancement: Ring enhancement was identified by the presence of a peripheral, circumferential zone of heightened contrast enhancement. (4) Enhancement direction: If he enhancement direction progresses radially from the hilum toward the periphery, it is referred to as centrifugal enhancement direction. Conversely, it is non-centrifugal enhancement direction.

**FIGURE 1:**
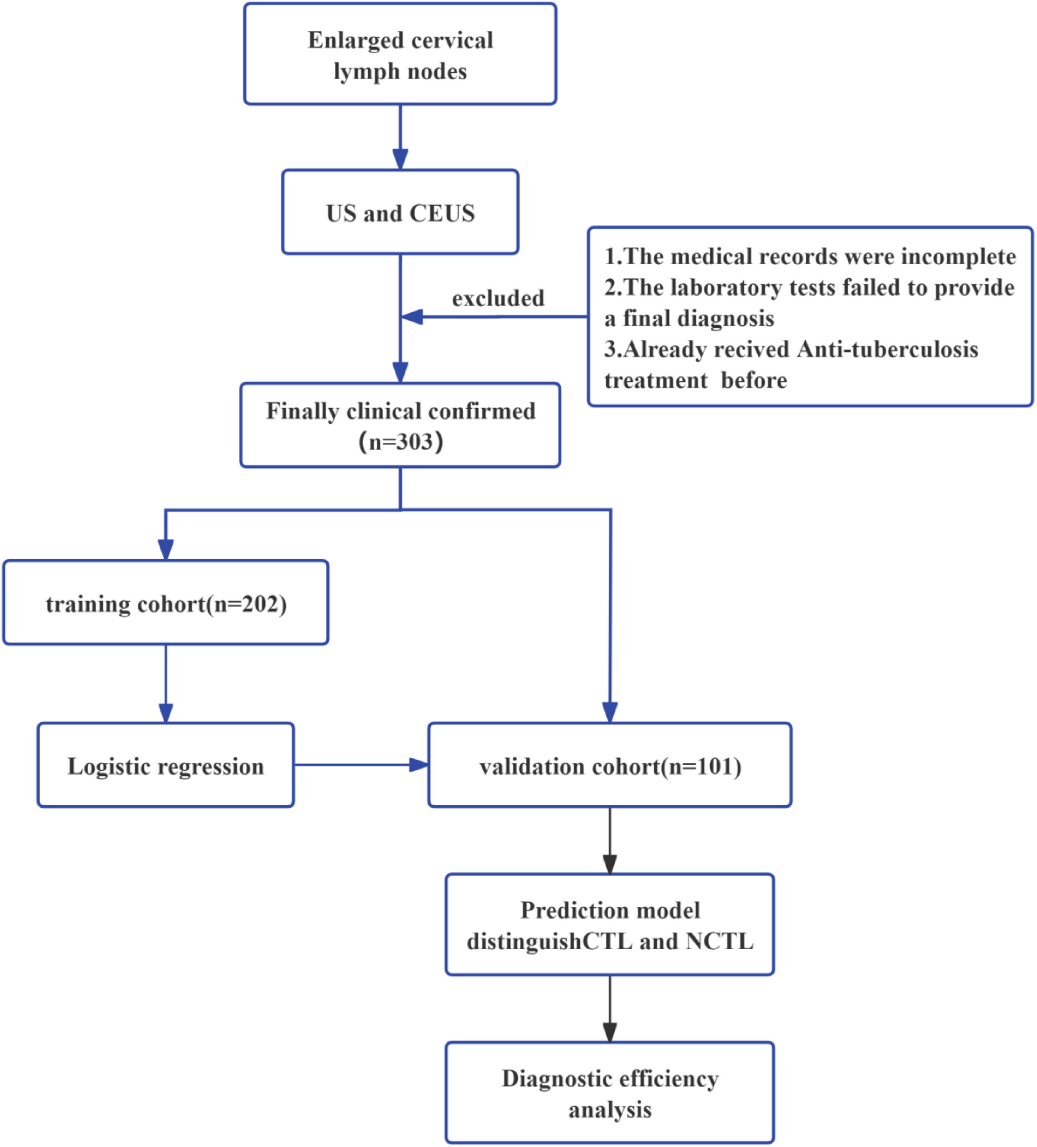
The flowchart.

Two ultrasound physicians, each with over 5 years of experience in superficial US, independently analyzed the images. In cases of discrepant interpretations, a senior physician was consulted to reach a consensus conclusion. Prior to reviewing the images, all ultrasound physicians were blinded to the final pathological diagnosis of the lymph nodes. The process of the study is shown in Figure 1.

### Statistical analysis

The SPSS statistical software package (version 23.0; SPSS Inc., Chicago, IL) was utilized for the univariate analysis of the qualitative features obtained from US and CEUS in the training cohort. The Chi-square test and Fisher’s exact test were applied to assess the differences in categorical data between the training and validation sets. For continuous data, the *t*-test was employed for analysis. A *p*-value of < 0.05 was considered statistically significant.

Various ultrasound multimodal features, such as the maximum longitudinal and transverse diameters, length-to-width ratio (L/S) of the lymph nodes, lymph node hilum, border definition, internal echogenicity, calcification, necrosis, blood flow distribution within the lymph nodes, and the direction of blood flow within the nodes, were subject to univariate analysis as independent variables. Statistically significant ultrasound features were selected and incorporated into a binary logistic regression analysis. Receiver operating characteristic (ROC) curves were constructed, with a significance level set at α = 0.05.

## Results

This study included 303 patients with cervical lymphadenopathy, comprising 164 males and 139 females, aged between 18 and 94 years, with a mean age of 46.3 ± 15.6 years. The overall samples were divided into the training cohort (202 cases) and the validation cohort (101 cases) at a ratio of 2:1. The training cohort consisted of 202 cases, including 113 males and 89 females, with an age range of 18–94 years and a mean age of 45.1 ± 18.7 years. Among them, 122 had TB, and 80 had non-tuberculous conditions (including 53 metastatic cases, 17 reactive hyperplasia cases, 8 lymphoma cases, and 2 cases of necrotizing lymphadenitis). The validation cohort comprised 101 patients, including 51 males and 50 females, aged between 18 and 82 years, with a mean age of 43.3 ± 17.1 years. Among them, 75 had TB, and 26 had non-tuberculous conditions (including 14 cases of lymphadenopathy, 10 metastatic cases, and 2 lymphoma cases). Baseline characteristics of the training and validation cohorts. Between cohort comparisons were performed using the *t*-test and χ^2^ test. *P* < 0.05 indicates statistical significance as shown in Table 1.

**TABLE 1:** The baseline characteristics of training cohort and validation cohort.

	Training cohort (*n* = 202)	Validation cohort (*n* = 101)	*t/*χ^2^	*P*
Mean age (years) (M ± SD)	45.1 ± 18.7	43.3 ± 17.1	0.839	0.402
Gender (female/male)	89/113	50/51	0.804	0.370
Short axis diameter			24.606	0.000
≥1.5 cm	117	28		
<1.5 cm	85	73		
L/S			0.245	0.621
≥2	120	57		
<2	82	44		
Margin			20.300	0.000
Clear	106	80		
Unclear	96	21		
Hilum			109.462	0.000
Absence	174	26		
Presence	28	75		
Echotexture			4.589	0.032
Homogeneous	38	30		
Hetergeneous	164	71		
Calcification			1.449	0.229
Absence	165	88		
Presence	37	13		
Necrosis			0.060	0.807
Absence	95	46		
Presence	107	55		
CDFI				
No detectable	19	12	0.449	0.503
Central	28	40	25.636	0.000
Peripheral	110	20	33.008	0.000
Mixed	45	29	1.511	0.219
Enhancement			0.000	1.000
Homogeneous	60	30		
Hetergeneous	142	71		
Perfusion defect			3.609	0.057
Absence	42	31		
Presence	160	70		
Peripheral ring enhancement			2.392	0.122
Absence	101	41		
Presence	101	60		
Enhancement direction			15.464	0.000
Non-centrifugal	130	41		
Centrifugal	72	60		

Ultrasonographic features included short axis diameter, the S/L ratio, boundary definition, presence of the lymph node hilum, internal echogenicity, calcification, necrosis, and color flow patterns. CEUS features included enhancement pattern, filling defects, peripheral ring enhancement, and enhancement direction.

The final clinical diagnosis was considered the dependent variable, where TB was coded as 1 and non-tuberculosis as 0. All aforementioned features were independent variables, with short axis diameter and the S/L ratio being continuous variables. Both S and the S/L ratio demonstrated a normal or near-normal distribution. Based on the arithmetic mean, thresholds were established for grouping: short axis diameter ( ≥1.5 cm, <1.5 cm) and S/L ratio ( ≥2, <2).

**FIGURE 2:**
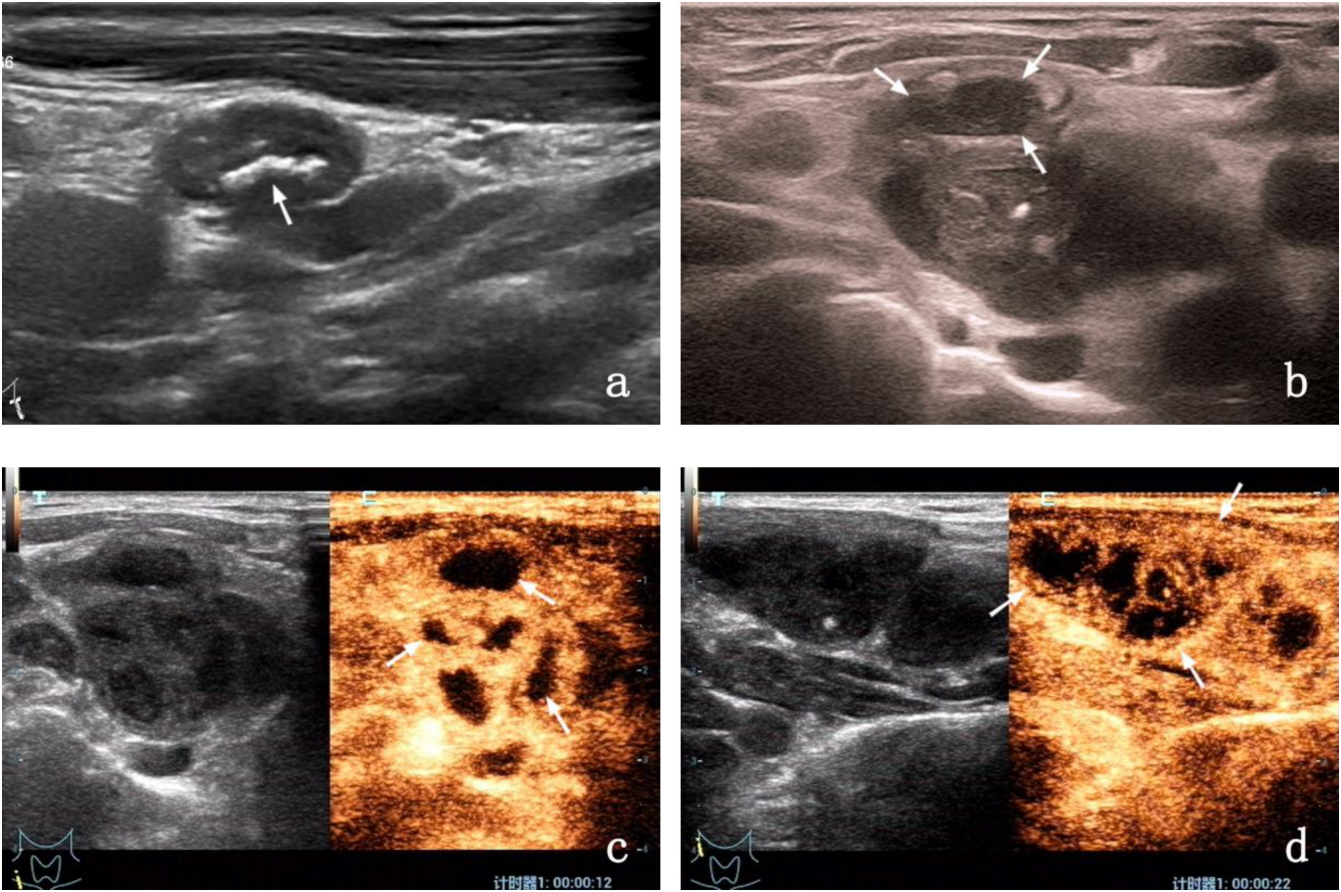
Images present general appearance of CTL for US and CEUS imaging patterns. (a) hilum absence (→). (b) hilum absence and internal Necrosis (→). (c) internal Hetergeneous enhancement and Perfusion defect (→). (d) internal Hetergeneous enhancement and peripheral ring enhancement (→).

The training cohort comprised 202 cases, of which 122 had TB and 80 had non-tuberculous conditions, as shown in Table 2 and Figure 2.

**TABLE 2:** Characteristics of US and CEUS for training cohort.

	CTL (*n* = 122)	NCTL (*n* = 80)	χ^2^	*P*
Short axis diameter			0.602	0.438
≥ 1.5 cm	68	49		
< 1.5 cm	54	31		
L/S			1.719	0.190
≥ 2	68	52		
< 2	54	28		
Margin			2.968	0.085
Clear	70	36		
Unclear	52	44		
Hilum			10.848	0.001
Absence	113	61		
Presence	9	19		
Echotexture			0.516	0.473
Homogeneous	21	17		
Hetergeneous	101	63		
Calcification			0.059	0.808
Absence	99	66		
Presence	23	14		
Necrosis			22.281	0.000
Absence	41	54		
Presence	81	26		
CDFI				
No detectable	13	6	0.565	0.452
Central	19	9	0.757	0.384
Peripheral	69	41	0.549	0.459
Mixed	21	24	4.563	0.033
Enhancement			17.369	0.000
Homogeneous	23	37		
Hetergeneous	99	43		
Perfusion defect			52.127	0.000
Absence	5	37		
Presence	117	43		
Peripheral ring enhancement			40.069	0.000
Absence	39	62		
Presence	83	18		
Enhancement direction			8.117	0.004
Non-centrifugal	88	42		
Centrifugal	34	38		

A multifactorial binary logistic regression model was constructed based on the US features (short axis diameter, the S/L ratio, boundary definition, presence of the lymph node hilum, internal echogenicity, calcification, necrosis, and color flow patterns) and CEUS characteristics (enhancement homogeneity, perfusion defects, peripheral ring enhancement, and enhancement direction) from the training cohort.

The statistically significant independent variables identified through univariate analysis, including the presence or absence of the lymph node hilum, necrosis, homogeneity of CEUS enhancement, peripheral ring enhancement, perfusion defects, and enhancement direction, were incorporated into a multivariate binary logistic regression model. The inclusion criterion for this model was set at *P* < 0.05.

The multivariate binary logistic regression analysis yielded the following predictive equation: Y = −6.658 + 2.222x_1_ + 0.817x_2_ + 2.491x_3_ + 1.094x_4_ + 2.382x_5_ + 1.481x_6_, where the constant is −6.658, and β represents the regression coefficient for each independent variable. In this equation, x_1_ denotes the lymph node hilum, x_2_ represents the presence of necrosis, x_3_ indicates whether the CEUS enhancement pattern is homogeneous, x_4_ refers to peripheral ring enhancement, x_5_ signifies perfusion defects, and x_6_ denotes the direction of contrast enhancement. The equation was subsequently applied to the validation cohort for diagnostic prediction, as detailed in Table 3.

**TABLE 3:** Multivariate analysis of binary logistic regression model.

Characteristic	β	*OR*	95%CI	*P*
Absence hilum	2.222	9.227	2.482–34.299	0.001
Necrosis	0.817	2.264	0.831–6.165	0.110
Hetergeneous enhancement	2.491	12.068	3.730–39.046	0.000
Peripheral ring enhancement	1.094	2.986	0.857–10.401	0.086
Perfusion defect	2.382	10.824	3.496–33.508	0.000
Not centrifugal enhancement direction	1.481	4.398	1.874–10.323	0.001
Constant	−6.658	0.001		

**FIGURE 3:**
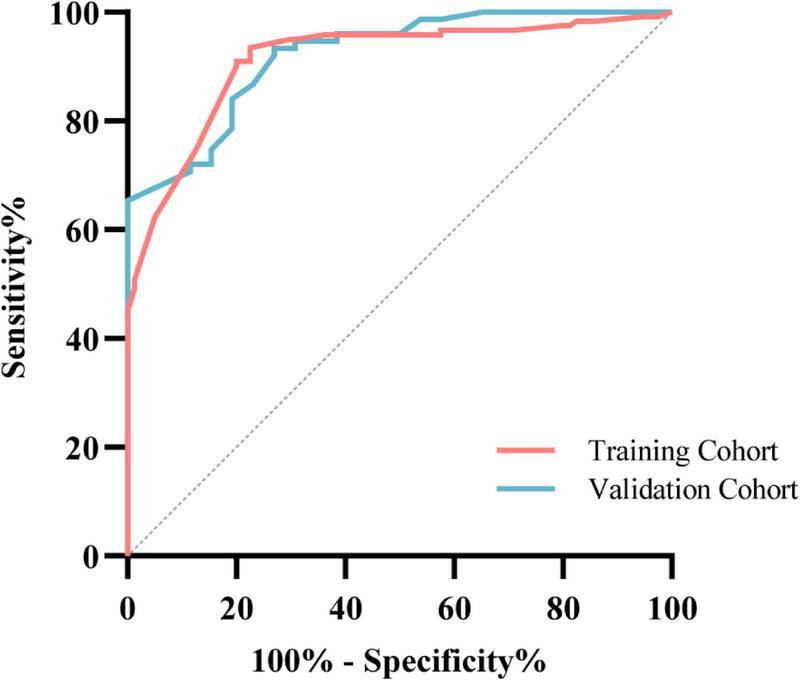
ROC curves for the training cohort and validation cohort.

In the training cohort, the area under the curve (AUC) for this regression model was 0.913, with a specificity of 77.5%, sensitivity of 92.6%, positive predictive value (PPV) of 86.2%, and negative predictive value (NPV) of 87.3%. In the validation cohort, the AUC was 0.917, with a specificity of 84.6%, sensitivity of 74.7%, PPV of 93.3%, and NPV of 53.7%. The corresponding ROC curves are presented in Figure 3.

## Discussion

Lymph node TB occurs when MTB invades the lymph nodes via the bloodstream, lymphatic system, or from adjacent infected sites, leading to chronic inflammation of the affected nodes (11). It can either be secondary to pulmonary TB or present independently, representing the most common form of extrapulmonary TB (12). When occurring independently, its clinical manifestations are often subtle, initially presenting only as enlarged cervical lymph nodes, which may be misdiagnosed as metastatic lymph nodes, lymphoma, or other conditions (13). Furthermore, US often fails to distinguish these conditions due to the absence of distinctive features. As a result, cervical lymph nodes may remain untreated until rupture occurs, leading to the formation of fistulas that are challenging to heal, thereby significantly compromising the patient’s quality of life (14, 15).

The pathological progression of LNTB is typically categorized into four stages: proliferative, necrotic, capsule rupture, and abscess formation (16). In the proliferative stage, the lymph nodes enlarge, form granulomas, harden, and remain mobile with mild tenderness. Following infection with MTB, lymphocytes and monocytes proliferate, forming granulomas. Ultrasound imaging often reveals enlarged lymph nodes with reduced echogenicity and uniform CEUS, although these images lack distinguishing characteristics. In the second stage, caseous necrosis develops within the lymph nodes, leading to the destruction of the lymph node hilum and surrounding caseous necrosis composed of epithelioid cells, lymphocytes, Langhans cells, and fibrous tissue. Ultrasound images display unclear internal structures with heterogeneous enhancement, which is a hallmark feature of LNTB on CEUS (17). The third stage is characterized by capsule rupture, resulting in perilymphatic inflammation and fusion of adjacent lymph nodes. During this phase, the nodes become immobile, with granulomas and chronic nonspecific inflammatory responses present both within and around the lymph nodes. Ultrasound typically demonstrates ill-defined boundaries and altered soft tissue echoes in the surrounding area. The final stage is marked by the combination of caseous necrosis and liquefactive tissue, leading to the formation of a tuberculous abscess. The abscess may rupture through the capsule, extending into subcutaneous or adjacent soft tissues, eventually forming a sinus tract that leads to skin ulceration (18).

In recent years, CEUS has gained widespread utilization in the diagnosis and management of CTL, significantly advancing both diagnostic imaging and therapeutic applications (19–22). In this study, we employed the second-generation contrast agent SonoVue, a blood pool agent encapsulated in a phospholipid shell containing sulfur hexafluoride. This agent effectively assesses blood perfusion in tissues and microcirculation within solid organs (8). Based on the perfusion characteristics of the contrast agent, CEUS imaging of CTL is generally categorized into three types: uniform enhancement, heterogeneous enhancement, and absence of enhancement. Studies have indicated that LNTB exhibits a larger area of enhancement on CEUS compared to US, while metastatic lymph nodes from papillary thyroid carcinoma show no significant size change post-enhancement (23).

Neither US nor CEUS provides a definitive diagnostic criterion for CTL. The multivariate binary logistic regression model, a statistical technique frequently employed in medical research, is used to assess the relationship between categorical dependent variables and independent variables, quantifying the influence of each predictor on the outcome (24, 25). Multi-modal ultrasound offers a more holistic evaluation of superficial LNTB (26). Hence, this study applied the multivariate binary logistic regression model to assess the diagnostic performance of ultrasound features in identifying cervical tuberculous lymphadenitis (CTL). Based on the US and CEUS characteristics, 12 features were selected as independent variables for regression analysis. After univariate regression analysis, several features were found to be statistically significant, including the visibility of the lymph node hilum, liquefactive necrosis, uniformity of enhancement pattern, perfusion defects, peripheral ring enhancement, and enhancement direction. These results align with previous studies (26, 27), which observed significantly higher rates of absent hilum (92.6% vs. 76.3%) and perfusion defects (95.9% vs. 53.8%) in CTL compared to non-tuberculous cervical lymphadenitis (NCTL). Furthermore, heterogeneous enhancement and peripheral ring enhancement were more prevalent in CTL, occurring in 81.1 and 56.6% of cases, respectively, compared to 52.5 and 22.5% in NCTL. The odds ratio (OR) for the heterogeneous enhancement pattern exhibited the highest absolute value, followed by CEUS perfusion defects, hilum disappearance, non-radial CEUS enhancement, peripheral ring enhancement, and necrosis, in descending order. These findings are consistent with the pathological development of LNTB, wherein caseous necrosis disrupts the hilum, leading to irregular blood supply and heterogeneous contrast agent perfusion. As granulomas form around the necrotic tissue, newly formed capillaries contribute to the peripheral ring enhancement observed on CEUS. Prior studies have also identified heterogeneous enhancement and peripheral ring enhancement as characteristic features of CTL, aligning with the present study’s findings (28). CEUS imaging revealed a significantly higher number of perfusion defects compared to necrosis observed on conventional US images, owing to CEUSosis observed on conto visualize lymph node blood perfusion. In normal lymph nodes, blood flow radiates from the hilum toward the periphery, following a centrifugal distribution pattern. However, lymph nodes affected by TB at various pathological stages exhibit differing enhancement patterns on CEUS, leading to potential variations in blood flow distribution. In cases of nodal lymphadenitis with exudates and surrounding inflammation, the associated pathological changes primarily involve peripheral lymphadenitis or tuberculous infiltration, often resulting in adhesions to surrounding tissues, while the hilum remains intact. Consequently, both the central and peripheral regions of the lymph node display blood flow signals simultaneously, yielding a mixed blood flow pattern or a non-centrifugal enhancement direction (29). In this study, AUC for the training and validation cohorts were 0.913 and 0.917. Some scholars have studied the use of logistic regression models to distinguish CTL from lymphoma. The AUC for the training and validation cohorts were 0.958 and 0.946 (30). Tong compared the performance of multimodal ultrasound and conventional ultrasound logistic regression models in differentiating between benign and malignant lymph nodes. The AUC of the MMUS model was 0.891, while that of the CUS model was 0.763 (31). Respectively, demonstrating robust discriminatory power and high decision reliability in diagnosing LNTB.

This study did have some limitations. First, the sample size was small and needs to be expanded to reduce sampling bias. Second, the clinical data were incomplete, limiting the comprehensiveness of the selected independent variables in the multivariate logistic regression model. Furthermore, CEUS imaging features may vary across different stages of CTL, which warranted further investigation to verify the diagnostic efficacy of each method and provide reliable theoretical support for clinical practice.

## Conclusion

In conclusion, the present study found that CEUS analysis, showing absent lymph node hilum, presence of liquefactive necrosis, heterogeneous enhancement, perfusion defects, peripheral ring enhancement, and non-centrifugal enhancement direction, demonstrated excellent predictive ability in distinguishing CTL from NCTL.

## Data Availability

The raw data supporting the conclusions of this article will be made available by the authors, without undue reservation.
